# Long‐Term Effect of Macrolides on *Helicobacter pylori* Eradication: Data From the European Registry on *Helicobacter pylori* Management (Hp‐EuReg)

**DOI:** 10.1111/hel.70107

**Published:** 2026-02-08

**Authors:** Olga P. Nyssen, Guillermo J. Ortega, Laimas Jonaitis, Ángeles Pérez‐Aísa, Bojan Tepes, Alfredo J. Lucendo, Javier Tejedor‐Tejada, Renate Bumane, Ana Garre, Jose M. Huguet, Monica Perona, Óscar Núñez, Manuel Pabón‐Carrasco, Manuel Castro‐Fernández, Miguel Areia, Jesús Barrio, Antonio Moreno Loro, Thomas J. Butler, María Soledad Marcos, Alma Keco‐Huerga, Manuel Domínguez Cajal, Maja Denkovski, Matteo Pavoni, György Miklós Buzás, Frode Lerang, Giuseppe Losurdo, Pablo M. Wolfe García, Perminder S. Phull, Samuel J. Martínez‐Domínguez, Juozas Kupcinskas, Mārcis Leja, Ricardo Marcos‐Pinto, Sinead M. Smith, Antonio Gasbarrini, Veronika Papp, Blas José Gómez Rodríguez, Mónica Sánchez Alonso, Ramón Pajares Villarroya, Pilar Pazo Mejide, Manuel Jiménez‐Moreno, Marta Pascual‐Mato, Concepción Bravo‐Pache, Milagrosa Montes, Anna Cano‐Català, Pablo Parra, Leticia Moreira, Francis Mégraud, Colm O’Morain, Luis Bujanda, Javier P. Gisbert, the Hp‐EuReg investigators, Jurij Bednarik, Eduardo Iyo, Fernando Bermejo, Virginia Flores, Miguel Fernández‐Bermejo, Ian L. P. Beales, Alisan Kahraman, Dan L. Dumitrascu, Ana Beatriz Pozo Blanco, Enrique Alfaro, Montserrat Planella, Victor A. Kamburov, Debora Compare, Natasa Brglez Jurecic, Georges Kamtoh, Antonietta G. Gravina, Benito Velayos, Eva Barreiro Alonso, Sabina Hrubá, Teresa Angueira, Piotr Szredzki, Melanija Razov Radas, Consuelo Ramírez, Noelia Alcaide, Christos Liatsos, Luis Fernández‐Salazar, Pedro Delgado Guillena, Sheyla Montori Pina, Piotr Eder, Jitka Vaculová, Jan Kral, Enrique Montil Miguel, Rosario Antón Ausejo, Gema Gigante González de la Aleja, Matteo Ghisa, Henrique Fernandes‐Mendes, Marinko Marušić, Stergios N. Kouvaras, Judith Gomez‐Camarero, Wojciech Marlicz, Deirdre McNamara, Pilar Mata‐Romero, María Badía Martínez, Miguel Suárez Matías, Inmaculada Ortiz‐Polo, Nayden Marinov Kandilarov, Benito Hermida Pérez, Sotirios D. Georgopoulos, Daniel Martin‐Holgado, Rosa Rosania, Alexander Link, Luis Hernández, Mila Kovacheva‐Slavova, Petra Čavajdová, Ramiro Carreño Macián, Andreas Blesl, Guillem Soy, Sergio Gil Rojas, David Přidal, Paola Chaudarcas, Giulia Fiorini, Pedro Almela, Anna‐Maria Tiefenthaller, Lumir Kunovsky, Saioa De la Maza Ortiz, Cristina Suárez Ferrer, Antonio Cuadrado, Francisco J. Rancel‐Medina, Diego Burgos‐Santamaría, Suzanne Cauchi, Isabel Pérez‐Martínez, Daniel Abad, Marko Nikolic, Teresa Valdés‐Lacasa, Lara Gassner, Riccardo Vasapolli, Natalie Friedova, Rosa M. Sáiz‐Chumillas, Tamara Matysiak‐Budnik, Petr Bauer, Noa Kapteijn, Jan Křivinka, Jorge Yebra Carmona, Gustavo Óliver Patrón‐Román, Marino Venerito, Manon C. W. Spaander, Carlos Rodríguez Pérez, Irene Blanco Bartolomé, Isabel Socorro Muñoz Hernández, Senador Moran Sanchez, Jan Bornschein Nuffield, Diego Ledro Cano, Pilar Bernal Checa, Antonio M. Caballero‐Mateos, Leticia Gimeno Pitarch, María de Lucas Gallego, Jakub Langner, Ángela Martínez Herreros, Patricia Sanz‐Segura, Melvyn Peña Gómez, Antonio Mestrovic, Gino Heeren, Adam Vasura, Patrick Dinkhauser, Mirjana Kalauz, Maria de los Ángeles Mejías Manzano, Petra Koňaříková, Jose Xavier Segarra Ortega, Karin Steidl, Alicia Granja Navacerrada, Edel Berroa de la Rosa, Raquel García‐Sánchez, Katja Repitsch, Antonio Díaz‐Sánchez, Monika Šindlerová, Jesus M. Gonzalez‐Santiago, Martin Schnierer, Laura Larrey Ruiz, Rodrigo Garcés‐Durán, Jesús Daniel Fernández‐de Castro, Irene Arteagoitia, Maria Fraile Gonzalez, Theodore Rokkas, Pierre Ellul, Lyudmila Boyanova, Antonia Perelló, Eduardo Albéniz

**Affiliations:** ^1^ Department of Gastroenterology Hospital Universitario de La Princesa, Instituto de Investigación Sanitaria Princesa (IIS‐Princesa), Universidad Autónoma de Madrid(UAM), and Centro de Investigación Biomédica en Red de Enfermedades Hepáticas y Digestivas (CIBERehd) Madrid Spain; ^2^ Unidad de Análisis de Datos Hospital Universitario de la Princesa, CONICET, Universidad Nacional de Quilmes–Argentina Madrid Spain; ^3^ Department of Gastroenterology Lithuanian University of Health Sciences Kaunas Lithuania; ^4^ Department of Gastroenterology Hospital Universitario Costa del Sol, RICAPPS Red de Investigación en Cronicidad, Atención Primaria y Prevención y Promoción de la Salud Marbella Spain; ^5^ Department of Gastroenterology DC Rogaska Slatina Slovenia; ^6^ Department of Gastroenterology Hospital General de Tomelloso, Instituto de Investigación Sanitaria Princesa (IIS‐Princesa), Centro de Investigación Biomédica en Red de Enfermedades Hepáticas y Digestivas (CIBERehd), Instituto de Investigación Sanitaria de Castilla‐La Mancha (IDISCAM) Tomelloso Spain; ^7^ Department of Gastroenterology Hospital Universitario de Cabueñes Gijón Spain; ^8^ Department of Gastroenterology Digestive Diseases Centre, Institute of Clinical and Preventive Medicine, University of Latvia Riga Latvia; ^9^ Department of Gastroenterology Hospital General Universitario de Valencia Valencia Spain; ^10^ Department of Gastroenterology Hospital Quirón Marbella Málaga Spain; ^11^ Department of Gastroenterology Hospital Universitario La Moraleja, Faculty of Medicine, Universidad Francisco de Vitoria, Faculty of Medicine, Universidad CEU San Pablo Madrid Spain; ^12^ Department of Gastroenterology Hospital Universitario Virgen de Valme Sevilla Spain; ^13^ Department of Gastroenterology Portuguese Oncology Institute of Coimbra, Faculty of Medicine of the University of Porto (FMUP), RISE@CI‐IPO (Health Research Network), Portuguese Oncology Institute of Porto (IPO Porto) Coimbra Portugal; ^14^ Department of Gastroenterology Hospital Universitario Río Hortega, Gerencia Regional de Salud de Castilla y León (SACYL) Valladolid Spain; ^15^ Department of Gastroenterology Hospital Universitario Virgen del Rocío Sevilla Spain; ^16^ Department of Gastroenterology, Clinical Medicine Trinity College Dublin, Tallaght University Hospital Dublin Ireland; ^17^ Department of Gastroenterology Hospital 12 de Octubre Madrid Spain; ^18^ Department of Gastroenterology Hospital Universitario Virgen Macarena Sevilla Spain; ^19^ Department of Gastroenterology and Hepatology Hospital Universitario San Jorge Huesca Spain; ^20^ Interni oddelek Diagnostic Centre Bled Slovenia; ^21^ Medical and Surgical Sciences Department Sant’Orsola‐Malpighi University Hospital Bologna Italy; ^22^ Department of Gastroenterology Ferencváros Health Center Budapest Hungary; ^23^ Østfold Hospital Trust Sarpsborg Norway; ^24^ Section of Gastroenterology, Department of Precision and Regenerative Medicine and Ionian Area University of Bari Bari Italy; ^25^ Department of Gastroenterology and Hepatology Hospital Sierrallana Torrelavega Spain; ^26^ Department of Digestive Disorders Aberdeen Royal Infirmary Aberdeen UK; ^27^ Department of Gastroenterology Hospital Clínico Universitario Lozano Blesa, Instituto de Investigación Sanitaria de Aragón (IIS Aragón), Centro de Investigación Biomédica en Red de Enfermedades Hepáticas y Digestivas (CIBERehd) Zaragoza Spain; ^28^ Gastroenterology Department Universidade do Porto, Centro Hospitalar do PortoInstituto De Ciências Biomédicas de Abel Salazar, Center for Research in Health Technologies and Information Systems (CINTESIS) Porto Portugal; ^29^ School of Medicine Trinity College Dublin Dublin Ireland; ^30^ Medicina Interna e Gastroenterologia Fondazione Policlinico Universitario Agostino Gemelli IRCCS Rome Italy; ^31^ Department of Surgery, Transplantation and Gastroenterology Semmelweis University Budapest Hungary; ^32^ Department of Gastroenterology Hospital Universitario Santa Bárbara Puertollano Spain; ^33^ Gastroenterology Section Hospital Universitario Infanta Sofía, Facultad de Medicina, Universidad Europea de Madrid Madrid Spain; ^34^ Department of Gastroenterology Hospital de Cruces Barakaldo Spain; ^35^ Gastroenterology Department Hospital Universitario de Burgos Burgos Spain; ^36^ Department of Gastroenterology and Hepatology, Clinical and Translational Research in Digestive Diseases Valdecilla Research Institute (IDIVAL), Marqués de Valdecilla University Hospital Santander Spain; ^37^ Centro de Salud María Auxiliadora (SERMAS) Madrid Spain; ^38^ Department of Microbiology Donostia University Hospital‐Biodonostia Health Research Institute San Sebastian Spain; ^39^ Gastrointestinal Oncology, Endoscopy and Surgery (GOES) research group Institut de Recerca i Innovació en Ciències de la Vida i de la Salut de la Catalunya Central (IRIS‐CC), Althaia Xarxa Assistencial Universitària de Manresa Manresa Spain; ^40^ Department of Gastroenterology Hospital Clínic de Barcelona, Centro de Investigación Biomédica en Red en Enfermedades Hepáticas y Digestivas (CIBERehd), Institut d’Investigacions Biomèdiques August Pi i Sunyer (IDIBAPS), University of Barcelona Barcelona Spain; ^41^ INSERM U1312, BRIC Université de Bordeaux Bordeaux France; ^42^ Department of Gastroenterology, Department of Medicine Biodonostia Health Research Institute, Universidad del País Vasco (UPV/EHU), Centro de Investigación Biomédica en Red de Enfermedades Hepáticas y Digestivas (CIBERehd) San Sebastián Spain

**Keywords:** antibiotic consumption, clarithromycin, eradication treatment, *H. pylori*, macrolide, resistance

## Abstract

**Background and Aims:**

Previous antibiotic use influences *Helicobacter pylori* antibiotic resistance. This study evaluated how prior population‐level macrolide (especially clarithromycin) use affects *H. pylori* eradication success in naïve patients.

**Methods:**

Retrospective, multicenter, ecological study. Multivariate logistic regression was performed with modified intention‐to‐treat effectiveness as the main outcome. Key variables included first‐line clarithromycin‐based treatments, therapy duration (7, 10, 14 days), proton pump inhibitor dose (low, standard, high), compliance (> 90%), and clarithromycin consumption (defined daily doses/1000 inhabitants/day, from the European Surveillance of Antimicrobial Consumption Network). Nested hierarchical models incorporated macrolide consumption, matched by year and country, and assessed the interaction between consumption and first‐line empirical treatments from the European Registry on *H. pylori* Management (Hp‐EuReg).

**Results:**

The study included 27,549 naïve patients from 23 countries with macrolide consumption data from 2013 to 2022. Higher macrolide consumption, within 0 to 8 years before treatment, was associated with reduced treatment effectiveness. The eradication rate consistently decreased as macrolide consumption increased, particularly within the previous 4 years. The efficacy of triple‐clarithromycin‐metronidazole, triple‐clarithromycin‐amoxicillin, and some bismuth‐quadruple therapies containing clarithromycin decreased with higher macrolide consumption. At the country level, higher population consumption of clarithromycin 2 years before treatment was associated with a decrease in eradication rates from 93% to 82%.

**Conclusion:**

Higher macrolide consumption in the general population negatively impacts the effectiveness of first‐line *H. pylori* regimens. These findings support that clarithromycin should only be administered as a susceptibility‐based therapy, with the strongest negative impact of prior population‐level exposure observed within 5 years and diminishing thereafter. ClincialTrials.gov number, NCT02328131.

**Abbreviations**
C: clarithromycin
DDD: defined daily dose
DID: daily dose per day per 1000 inhabitants
ESAC‐Net: European surveillance of antimicrobial consumption network
Hp‐EuReg: European registry on *Helicobacter pylori* management

*H. pylori*

:
*Helicobacter pylori*

LRT: likelihood ratio test
mITT: modified intention to treat
OR: odds ratio

## 1 Introduction

*Helicobacter pylori* ( *H. pylori* ) infects over 40% of the global population [1], causing symptoms or disease in 10% of the cases, potentially impacting 800 million people [2]. It commonly leads to dyspepsia but can also cause severe conditions like gastric cancer [3, 4, 5].

*Helicobacter pylori* ( *H. pylori* ) infection is challenging due to limited effective antibiotics and rising resistance. Commonly used clarithromycin‐based therapies [6] lose efficacy when resistance exceeds 15%, as shown in several studies, particularly when empirical (non–susceptibility‐based) therapies are used [7, 8, 9]. Based on these data, current guidelines discourage its use beyond this threshold [4, 5], and clarithromycin should only be administered as a susceptibility‐based therapy.

Rational antibiotic use is crucial in preventing microbial resistance [10], and a clear relationship exists between antibiotic consumption and *H. pylori* resistance [11, 12, 13, 14, 15, 16, 17]. However, it remains unclear whether prior antibiotic use in the general population impacts the effectiveness of *H. pylori* eradication treatments.

In this ecological study, our objective was to analyze the relationship between the population level of antibiotic consumption (by year and country) in Europe and the effectiveness of eradication treatments in individual treatment‐naïve patients infected with *H. pylori* .

## 2 Methods

Antibiotic consumption data were obtained from the European Centre for Disease Prevention and Control (ECDC) through the European Surveillance of Antimicrobial Consumption Network (ESAC‐Net), which tracks antibiotic use (J01 group of the anatomic therapeutic chemical (ATC) classification) [10] in the community and hospitals. Antibiotic use is measured in daily dose (DDD) per 1000 inhabitants per day (DID) [18]. These data exclude antibiotic use in animal husbandry and fish culture.

*Helicobacter pylori* ( *H. pylori* ) treatment efficacy data for naïve patients were sourced from Hp‐EuReg. Hp‐EuReg is a multicenter, non‐interventional registry (38 countries) started in 2013 by the European Helicobacter and Microbiota Study Group (www.helicobacter.org) [6, 18]. The study follows ethical guidelines, was approved by the Hospital Universitario de La Princesa Ethics Committee, and is registered on ClinicalTrials.gov (NCT02328131). Further information is detailed in the published protocol [19].

### 2.1 Data Management

Hp‐EuReg data were recorded in an Electronic Case Report Form (e‐CRF) at REDCap hosted at “Asociación Española de Gastroenterología” (AEG; www.aegastro.es) [20, 21], anonymized, and included first‐line treatments registered between June 2013 and June 2023. Written informed consent was obtained from all participants. The data set was restricted up to 2022 to align with ESAC‐Net records.

After data extraction, the Hp‐EuReg data were quality‐reviewed and discordances were resolved by querying the investigators.

The data collection from the ESAC‐Net database was evaluated for validity using a checklist addressing various biases, including coverage bias in census data, sampling bias in sample data, unaccounted over‐the‐counter sales in reimbursement data, issues with parallel trade or inadequate registration of non‐reimbursed antibiotics, and shifts in antibiotic use between ambulatory and hospital care. To ensure comparability, results were standardized as Defined Daily Doses (DDD) per 1000 inhabitants per day (DID), with population estimates based on mid‐year figures from the WHO European Health for All database [22]. A comprehensive methodology description, including data provider details and discussions on validity, is available in a separate publication [23].

### 2.2 Variable Categorization and Definitions

To enhance data interpretation, variables were categorized. Proton pump inhibitor (PPI) doses were standardized to omeprazole equivalents and grouped as low‐dose (4.5–27 mg of omeprazole equivalents given twice a day), standard‐dose (32–40 mg of omeprazole equivalents given twice a day), and high‐dose (54–128 mg of omeprazole equivalents given twice a day) [24, 25].

Treatment durations were classified as 7, 10, or 14 days. Compliance was defined as ≥ 90% of medication taken. Only empirically treated patients were included. Seven treatment regimens were analyzed. Those including macrolides were: Triple‐CA (PPI, clarithromycin, amoxicillin); Triple‐CM (PPI, clarithromycin, metronidazole); Seq‐CAT‐CAM and Conco‐CAT‐CAM (PPI, clarithromycin, amoxicillin, metronidazole or tinidazole); Quad‐CAB (PPI, clarithromycin, amoxicillin, bismuth). Regimens without macrolides were: BQT (PPI, metronidazole or tinidazole, tetracycline, and bismuth) and Sc‐BQT (PPI, metronidazole, tetracycline, and bismuth prescribed as a three‐in‐one single capsule).

Effectiveness was evaluated using a modified intention‐to‐treat (mITT) analysis, including all patients empirically treated (not receiving a susceptibility‐guided antibiotic prescription) who completed follow‐up. *H. pylori* eradication was confirmed ≥ 4 weeks post‐treatment using urea breath test, stool antigen, or histology.

### 2.3 Data Analysis

A logistic regression model was initially constructed to assess the effectiveness of different *H. pylori* eradication treatments. The explanatory variables included compliance (Yes/No), treatment duration (7, 10, or 14 days), PPI dose (low, standard, or high), and treatment type (seven categories: Triple‐CA, Triple‐CM, Conco‐CAT‐CAM, Seq‐CAT‐CAM, Quad‐CAB, BQT, and Sc‐BQT). The outcome variable was treatment success or failure based on the mITT analysis. BQT and Sc‐BQT, which do not contain macrolides, were used as the reference category for treatment comparison.

To refine the model, an Akaike step‐backward selection was performed, but none of the variables could be removed without compromising model performance. The Akaike Information Criterion (AIC) was used to balance model fit and complexity, with lower AIC values indicating better optimization.

To evaluate the potential ecological association between community‐level macrolide consumption and clarithromycin‐based treatment effectiveness, macrolide consumption data (mean: 2.77; range: 0.58–7.88) were incorporated in the regression model. A country‐ and year‐matched approach was applied to align macrolide consumption values with patient treatments. The analysis explored whether past macrolide consumption influenced treatment outcomes using three approaches:

- Annual consumption data from previous years (1–10 years before treatment, covering 2013–2022).
- Cumulative consumption over 1–10 years (2013–2022).
- Average macrolide consumption during the same period (2013–2022).

A delay of zero years was applied for patients who received treatment in the same year as macrolide consumption, regardless of whether the antibiotics were taken before or after treatment.

To assess the interaction between macrolide consumption and clarithromycin‐based treatments, an interaction term was included in the regression model. This interaction was evaluated through the ratio of two odds ratios (OR). The first OR compared clarithromycin‐based treatments to non‐clarithromycin‐based treatments at a given value of macrolide consumption, a continuous variable. The second OR examined the same comparison but with macrolide consumption increased by one unit. The resulting ratio, usually referred to as the OR of the interaction, indicated whether macrolide consumption influenced treatment effectiveness; an OR less than one suggests that increased macrolide consumption is associated with reduced effectiveness of clarithromycin‐based treatments, indicating a potential negative impact. Conversely, an OR greater than one implies a potentiation effect, where higher macrolide consumption may enhance the effectiveness of these treatments.

**FIGURE 1:**
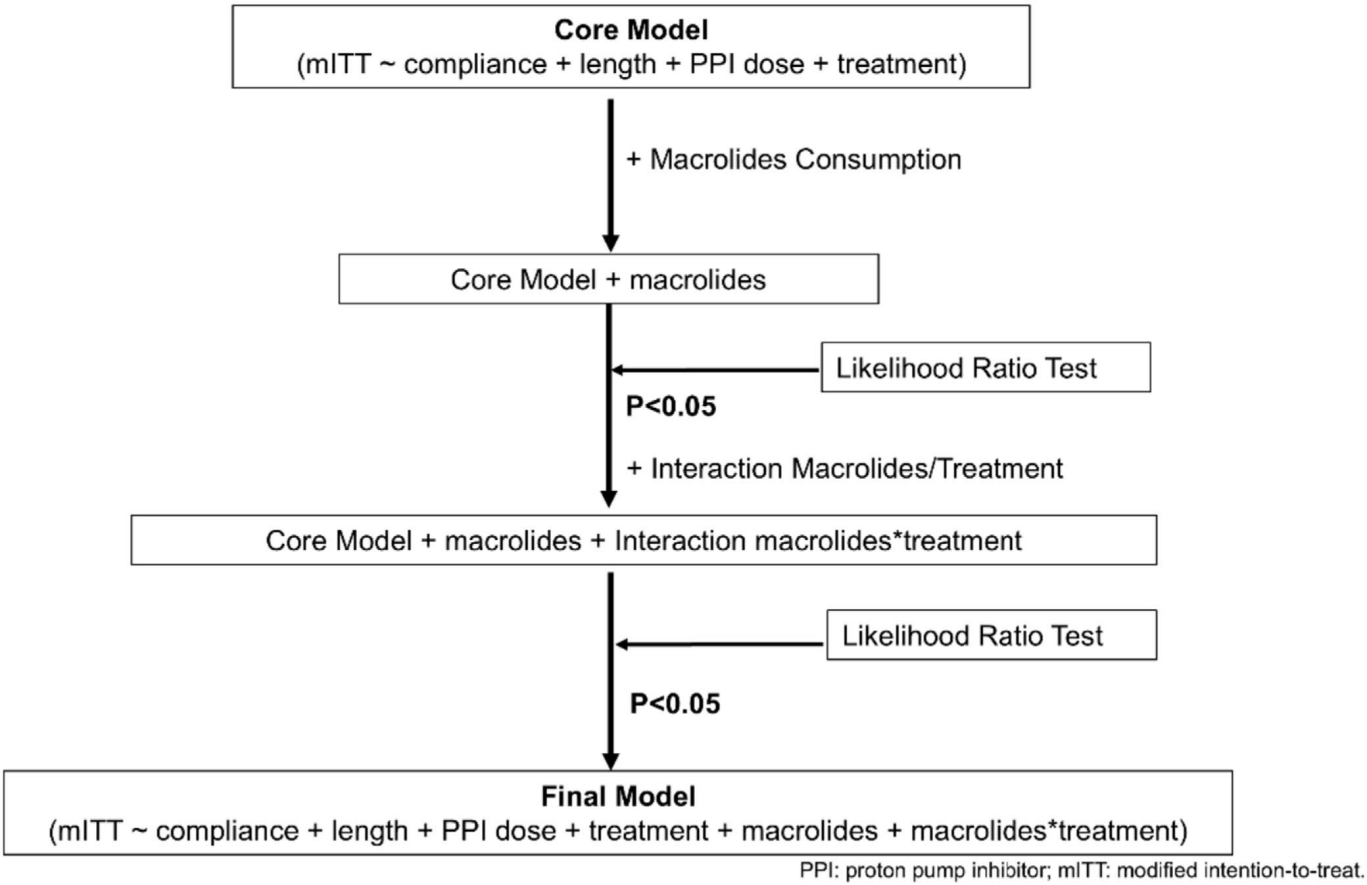
Hierarchical models. The flow chart depicts the construction of three models used in this study: the core model, the model with macrolide consumption, and the model with macrolide consumption–treatment interaction. mITT, modified intention‐to‐treat effectiveness; PPI, proton pump inhibitor.

Model improvement was evaluated using the likelihood ratio test (LRT), which compared nested models to determine whether additional predictors significantly enhanced the model fit. The first step involved adding macrolide consumption to the core model, followed by evaluating its impact with the LRT. If this addition improved the model, an interaction term was subsequently introduced, and its significance was also tested again with LRT (Figure 1).

Qualitative variables were reported as relative frequencies, expressed as percentages with 95% confidence intervals (CIs). Statistical significance was defined as *p* < 0.05. All analyses were conducted using R version 4.1.2, with custom scripts and base functions from the R programming environment (http://www.R‐project.org; the R Foundation for Statistical Computing, Vienna, Austria) [26].

## 3 Results

**FIGURE 2:**
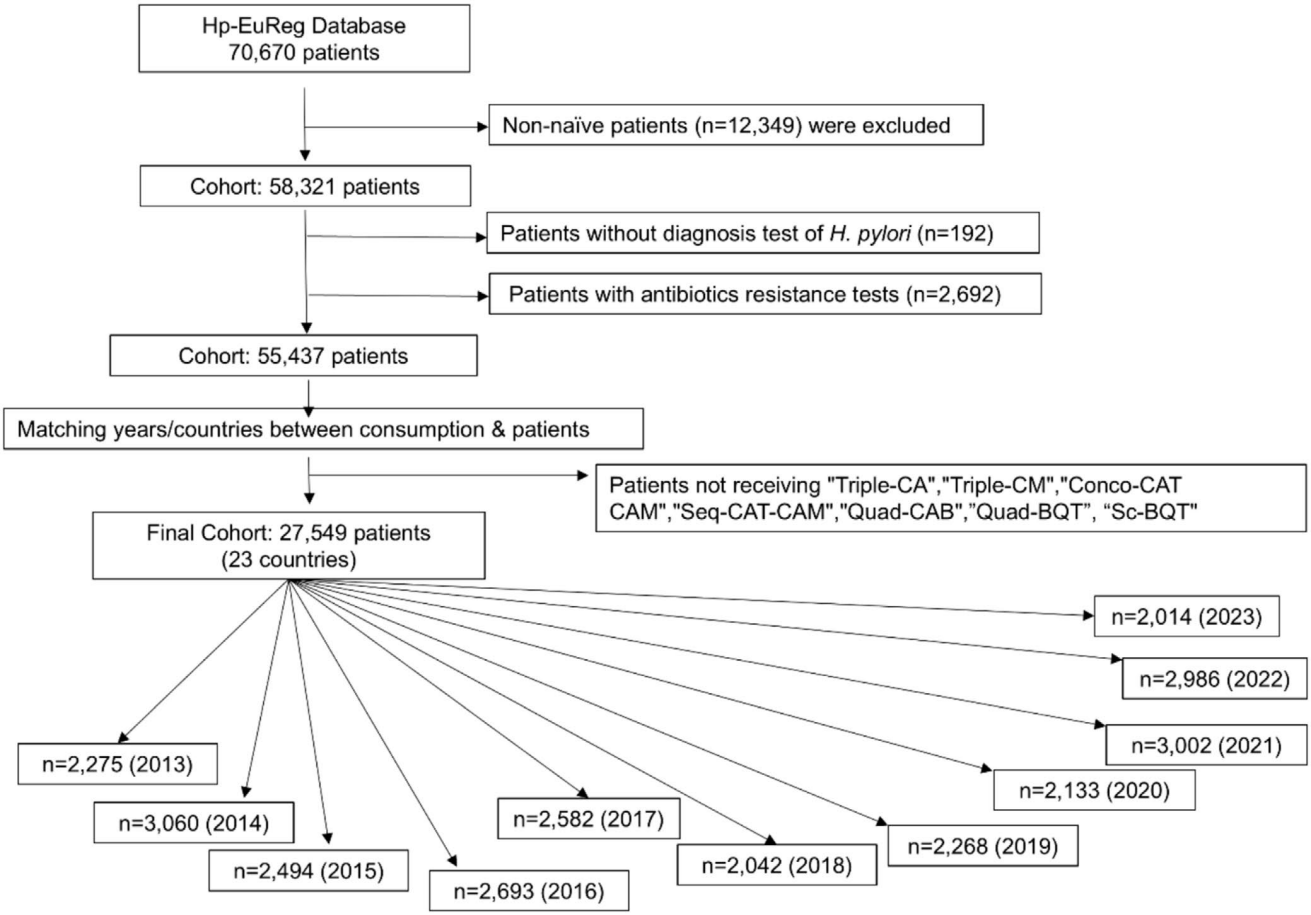
Flow chart of patient selection. A, amoxicillin; B, bismuth; BQT: Bismuth quadruple therapy prescribed with metronidazole, tetracycline and bismuth administered separately; C, clarithromycin; M, metronidazole; n, number of treatment‐naïve patients prescribed with an empirical therapy; Sc‐BQT, bismuth quadruple therapy prescribed with metronidazole, tetracycline and bismuth all administered in a single capsule (Pylera); T, tinidazole; PPI, proton pump inhibitor.

A subset of 58,321 treatment‐naïve patients was first selected from the original Hp‐EuReg database (70,670 patients enrolled between 2013 and 2023). From this group, a further refined subsample of 27,549 patients was chosen for analysis based on their treatment regimen. Patients included in the final analysis had received one of the following seven first‐line eradication treatments: Triple‐CA, Triple‐CM, Conco‐CAT‐CAM, Seq‐CAT‐CAM, Quad‐CAB, BQT, and Sc‐BQT (Figure 2). These patients were recruited from 23 European countries, with the highest number of participants coming from Spain (*n* = 16,517), followed by Italy (*n* = 3247), and Slovenia (*n* = 2889). The patient distribution across the remaining countries covered Western, Northern, Southern, Central, and Eastern Europe, as well as the Baltic region.

### 3.1 Global Effect of Macrolide Consumption on Treatment Effectiveness

**FIGURE 3:**
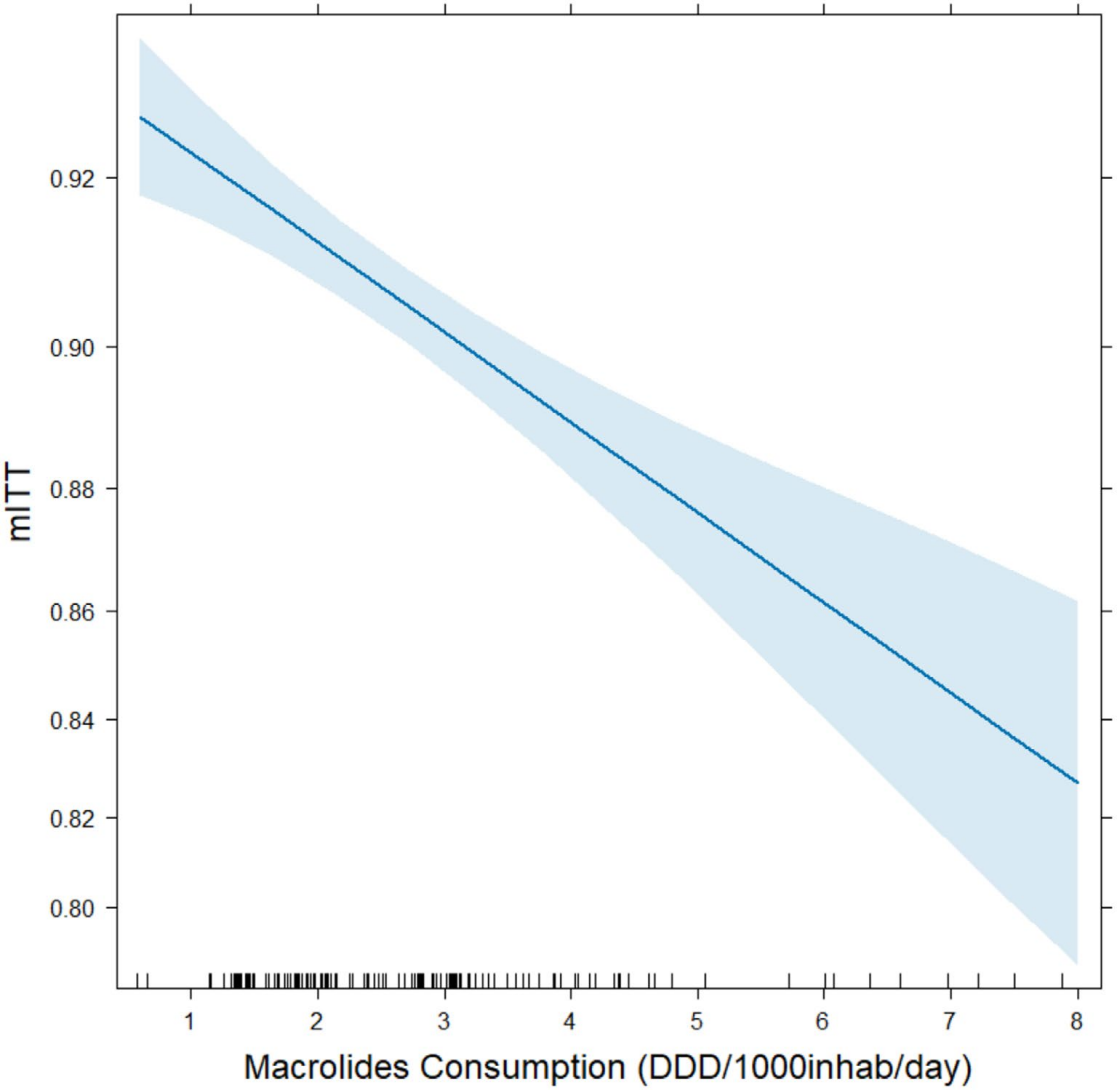
Effect on the modified intention‐to‐treat effectiveness of macrolide community consumption a year before clarithromycin‐based treatment. DDD, defined daily dose (expressed as number/1000 inhabitants/day); mITT, modified intention to treat effectiveness.

The impact of macrolide consumption on treatment effectiveness was analyzed by adding this variable as covariate in the core model. Model performance improved significantly when macrolide consumption was introduced (LRT *p* = 3.84 × 10^−7^). The analysis of 23,131 patients across 21 countries revealed that clarithromycin‐based treatments became less effective with increased macrolide consumption in the previous year (Figure 3). The effect of macrolides was a reduction of treatment effectiveness of 13%.

**FIGURE 4:**
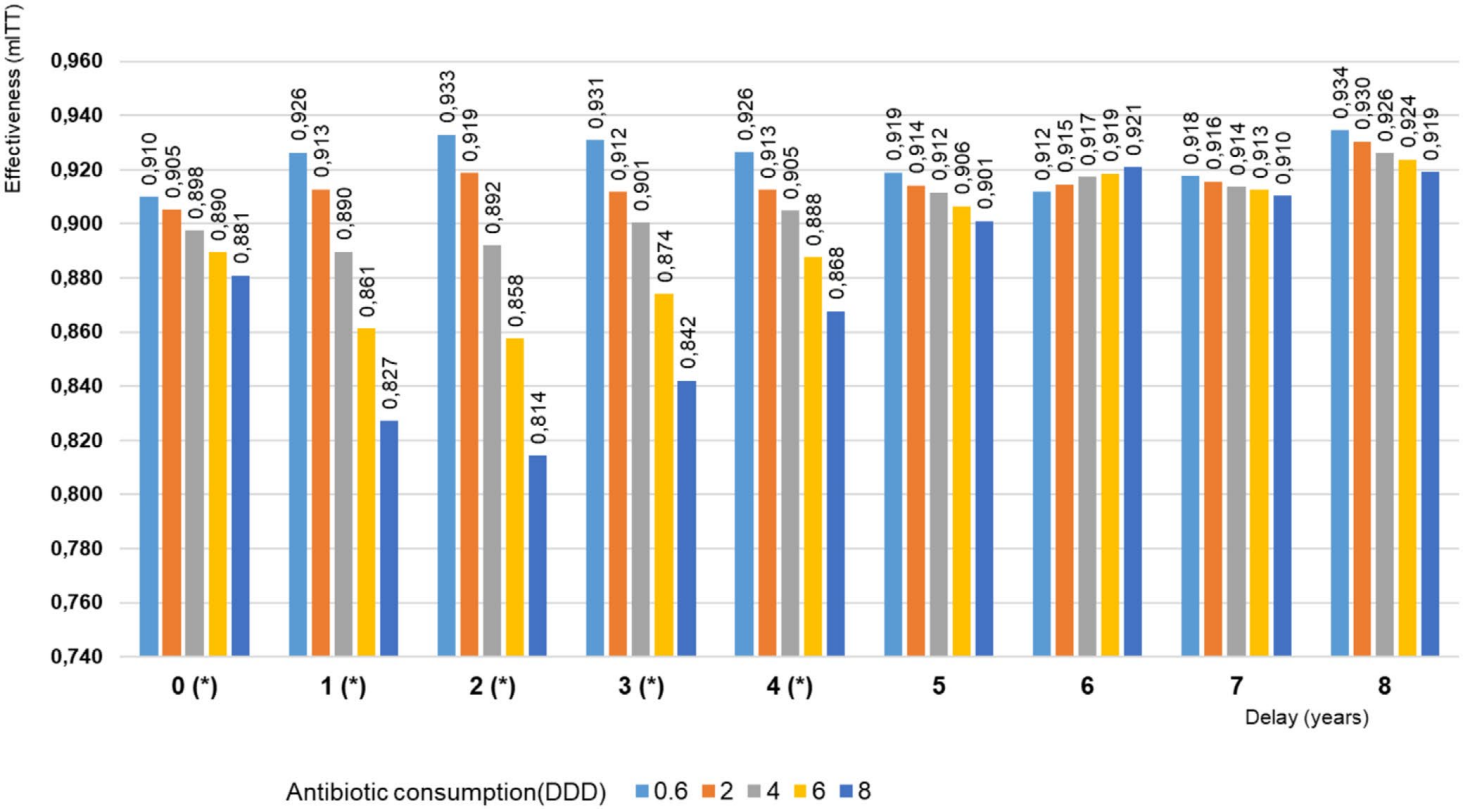
Effect of macrolide community consumption on clarithromycin‐based treatment effectiveness for different delay periods between consumption and treatment. Different consumption levels (0.6, 2, 4, 6, 8 DDD/1000 inhabitants/day) are color‐coded. The x‐axis represents delay times between consumption and treatment year. Treatment effectiveness by modified intention to treat (mITT) was analyzed. *Significant model improvement with a likelihood ratio test (LRT), *p* < 0.05. DDD, defined daily dose.

We analyzed the effect of macrolide consumption on mITT effectiveness at different time delays before treatment (Figure 4). For each added year of delay, marginal effects were assessed at five macrolide consumption values. The core models with and without this variable were compared by LRT. Effectiveness decline was most pronounced within the first 2 years after consumption, peaking at 3 years before beginning to recover at four‐ and five‐year delays.

When countries with the highest number of reported cases were excluded—Spain alone (59%); Spain and Italy (72%); or Spain, Italy, and Slovenia (82%) (Figure S1)—the overall pattern did not change substantially. However, across all three sensitivity analyses, a negative association between antibiotic consumption and resistance emerged during the first 3 years, in contrast to the global model where the resistance peak appeared after a two‐year lag. Moreover, a shift in treatment efficacy was observed toward the fourth year. Notably, for the entire series from 0 to 8 years, the model excluding Spain (the country with the highest number of cases) alone consistently converged—that is, it consistently supported the inclusion of macrolide use—whereas the original model ceased to converge from the fifth year onward. Similar results were obtained when excluding patients from Spain and Italy, or from Spain, Italy, and Slovenia.

### 3.2 Interactions Between Macrolide Consumption and Treatments

To further assess the impact of macrolide consumption on mITT effectiveness, an interaction term between macrolide consumption and treatment was added to the core model (Figure 1). LRT analysis confirmed that including these terms improved model fit across all time delays (Table 1). For one‐year delay, the treatments most affected were Triple‐CA (OR: 0.80, p: 4.5 × 10^−3^), Triple‐CM (OR: 0.50, p: 1.69 × 10^−5^), and Quad‐CAB (OR: 0.55, p: 1.39 × 10^−2^). Increasing macrolide consumption by one unit reduced Triple CA effectiveness by 20% (OR: 0.8). Similar patterns were observed for delays up to 8 years, improving the fit of the model at all the time delays (Table 2). (Table 3) presents the effects of the interaction macrolide consumption/treatment on treatment effectiveness for delays of 0–8 years. For Triple‐CA, the interaction with macrolide consumption was significant across most delays. ORs were below one, indicating that higher levels of macrolide consumption were associated with reduced effectiveness compared to non‐clarithromycin‐based treatments. Similar patterns were observed for Triple‐CM, throughout different delays.

**TABLE 1:** Effect on treatment effectiveness of the interaction between treatments and macrolide consumption in the community 1 year before treatment. The table presents odds ratios, confidence intervals, and significance for a logistic regression model assessing macrolide consumption (previous year), treatment duration, PPI dose, regimen, and eradication outcome. Reference levels were Sc‐BQT and BQT (7 days), low‐dose PPI, and non‐compliance.

	OR	2.50%	97.50%	*p*
(Intercept)^a^	1.8039	1.1194	2.9039	1.53E‐02*
Compliance	8.8386	6.9731	11.1915	5.32E‐73***
Length10 days	0.7564	0.6377	0.8957	1.28E‐03**
Length14 days	0.8715	0.7288	1.0404	1.30E‐01
Standard‐dose PPI	1.7664	1.5519	2.0150	1.32E‐17***
High‐dose PPI	2.0250	1.8153	2.2608	2.01E‐36***
Triple‐CA	0.5869	0.3800	0.9070	1.63E‐02*
Triple‐CM	0.8806	0.4166	1.8697	7.40E‐01
Conco‐CAT CAM	0.5119	0.3238	0.8083	4.11E‐03**
Seq‐CAT‐CAM	0.8371	0.3140	2.2303	7.22E‐01
Quadruple‐CAB	2.0910	0.5973	7.6683	2.56E‐01
Macrolides	0.9604	0.8419	1.0991	5.52E‐01
Triple‐CA:Macrolides	0.7990	0.6836	0.9318	4.50E‐03**
Triple‐CM:Macrolides	0.4921	0.3558	0.6801	1.69E‐05***
Conco‐CAT CAM:Macrolides	1.0648	0.9083	1.2476	4.38E‐01
Seq‐CAT‐CAM:Macrolides	0.9517	0.7354	1.2345	7.08E‐01
Quad‐CAB:Macrolides	0.5561	0.3471	0.8856	1.39E‐02*
Signif. codes: 0 ‘***’ 0.001 ‘**’ 0.01 ‘*’ 0.05 ‘ ’ 0.1 ‘ ’ 1				

Abbreviations: A, amoxicillin; B, bismuth; C, clarithromycin; M, metronidazole, OR, odds ratio; PPI, proton pump inhibitor; T, tinidazole.

^a^ The ‘intercept’ represents the baseline log odds of treatment success for the reference group. Compliance is entered next to emphasize its substantial contribution to eradication outcomes. Scientific notation (e.g., 1.53E‐02) denotes exponential values (1.53 × 10^−2^).

**TABLE 2:** Likelihood Ratio Test for different logistic regression models throughout the full range of delays between macrolide consumption and treatment.

	*p* value of LRT including macrolides	*p* value of LRT including macrolides/treatment interaction	*n*° Patients	*n*° Countries	Years
Same year	0.07294	1.848e‐14	25,525	23	10
1 year‐delay	3.843e‐07	1.155e‐13	23,131	21	9
2 year‐delay	1.782e‐08	8.944e‐16	19,935	19	8
3 year‐delay	7.271e‐06	2.103e‐09	17,381	16	7
4 year‐delay	0.002985	1.008e‐08	14,683	16	6
5 year‐delay	0.3623	2.2e‐16	12,106	16	5
6 year‐delay	0.6422	2.209e‐14	9992	16	4
7 year‐delay	0.7378	3.058e‐05	7882	15	3
8 year‐delay	0.4899	6.207e‐05	5850	16	2

*Note:* The table presents LRT (Likelihood Ratio Test) results comparing the core model with macrolide consumption alone and with interaction terms, showing significant improvements. Non‐significant results are highlighted in gray.

**TABLE 3:** Effect on treatment effectiveness of the interaction between *H. pylori* eradication treatments and macrolide consumption in the community throughout the full range of delays between macrolide consumption and treatment.

	Years of delay								
	0	1	2	3	4	5	6	7	8
Triple‐CA:Macrolides	0.65	0.80	NS	NS	0.76	0.61	0.56	0.72	0.63
Triple‐CM:Macrolides	0.35	0.50	0.51	0.55	NS	0.60	0.57	NS	NS
Conco‐CAT CAM:Macrolides	NS	NS	1.25	1.18	NS	NS	NS	NS	NS
Seq‐CAT‐CAM:Macrolides	0.78	NS	NS	NS	NS	1.18	NS	NS	NS
Quad‐CAB:Macrolides	NS	0.56	0.53	NS	NS	6.44	NS	88,000	0.009

*Note:* The table displays significant (< 0.05) odds ratios for interaction terms in macrolide‐based treatments across different delays.

Abbreviations: A, amoxicillin; B, bismuth; C, clarithromycin; M, metronidazole; NS, non‐significant interactions; T, tinidazol.

Conversely, ORs for Conco‐CAT‐CAM were slightly above one for two‐ and three‐year delays, as was that of Seq‐CAT‐CAMat for the five‐year delay, though effects were clinically minor. Quad‐CAB showed reduced effectiveness for one‐ and two‐year delays, but extreme OR values (6.44 and 88,000) for longer delays, likely due to small sample sizes affecting model stability. In fact, the last four cases for this treatment (5‐, 6‐, 7‐, and 8‐year delays) were calculated with 7.3%, 4%, 3.6%, and 3.5% of cases with respect to the reference level, highlighting a clear lack of robustness due to model instability.

Despite these variations, results remained consistent in the analysis of data from all countries and from those with the largest patient samples (Spain and Italy), thus reinforcing the reliability of the findings.

### 3.3 Average and Accumulated Macrolide Consumption

Macrolide consumption was analyzed as both accumulated and average intake over the years, but neither approach improved the core model, as LRT analysis showed no statistically significant effect.

## 4 Discussion

In the current study, we observed that previous macrolide consumption at the population level was ecologically associated with a significant reduction in the effectiveness of clarithromycin‐containing therapies in individual patients.

Several studies link personal antibiotic use to resistance and reduced efficacy [27, 28]. Accordingly, some authors recommend against clarithromycin‐based regimens after prior exposure [27], while others highlight the benefit of optimizing treatments based on past macrolide use [29].

Our study found that the effectiveness of clarithromycin‐based treatments was influenced not just by whether clarithromycin was used, but also by the DDD amount. A linear relationship was observed, with eradication rates declining as the daily dose increased. Specifically, eradication rates dropped from 93% for consumption below 1 DDD to 82% for 8 DDD. We also unveiled two key findings: firstly, higher levels of prior macrolide consumption were linked to lower treatment effectiveness, particularly if consumption occurred within 4 years before treatment; secondly, the negative impact was more pronounced for consumption within 2 years prior to receiving treatment and at higher macrolide doses.

Studies on other infections have shown that antibiotic resistance is higher when exposure occurs closer to treatment [30, 31], and decreases gradually over time [32, 33]. This pattern has been observed in both primary care and hospital settings [31, 34]. In hospitals, resistance often develops within 0 to 6 months after exposure [34]. For macrolides, resistance can persist for at least three months [30].

In our study, the global effects—analyzed with the core model including macrolide consumption but not the interaction between macrolide consumption and treatment—remained significant for up to a four‐year delay. Although adding macrolide consumption from the fifth year onward did not improve the core model's fit, the results still followed a similar pattern to earlier years, except for the six‐year delay. This pattern suggested that higher antibiotic consumption remained associated with lower treatment effectiveness over an extended period, though with a weaker impact. Previous studies suggest that longer intervals between exposure and treatment reduce the negative impact on *H. pylori* eradication [13, 14]. This may be due to interactions among diverse *H. pylori* colonies within a patient, leading to the disappearance of resistant strains. However, research beyond 5 years is limited [15], and longitudinal studies tracking strain evolution are lacking. Clarithromycin resistance results from irreversible mutations in the 23S rRNA gene, the antibiotic's binding site, with A2143G, A2142G, and A2142C being the most common mutations worldwide [12, 35]. Triple therapies were most affected by prior antibiotic use, with Triple‐CM and Triple‐CA showing a 50% and a 20% reduction, respectively. It is reasonable to assume that triple therapies involving antibiotics with higher antibiotic resistance rates, such as clarithromycin (over 15%) and metronidazole (over 30%), are more directly influenced by previous antibiotic consumption; thus, the greater susceptibility of Triple‐CM (OR = 0.50) to macrolide exposure compared to Triple‐CA (OR = 0.80) might stem from potential synergistic resistance between clarithromycin and metronidazole. Regional resistance data may help clarify this interaction as the decline persisted in the analysis of longer delays, especially for Triple‐CM. Interestingly, Quad‐CAB also showed reduced effectiveness with prior clarithromycin use, suggesting that even with bismuth, clarithromycin resistance remains influential. Additionally, Extreme OR values, such as ORs 88,000 or 0.009 for Quad‐CAB at 7 and 8‐year delay, reflect model instability due to small samples. These were retained for transparency but should be interpreted cautiously. Missing values in Table 3, such as for Triple‐CA and Triple‐CM in 2–4 years, reflect strata non‐significant interactions. Interestingly, for Conco‐CAT‐CAM, ORs were slightly above one for 2‐ and 3‐year delays, raising the possibility that concomitant therapy may be less susceptible to the effects of prior clarithromycin exposure. This finding contrasts with the treatment's exclusion in recent ACG guidelines and warrants further investigation in future trials.

Our study has several limitations. Firstly, due to its retrospective nature, several variables that may influence outcomes, such as smoking, alcohol consumption, socioeconomic status, hygiene practices, use of concomitant medications, and body mass index, among others, could not be analyzed. Antibiotic consumption was not assessed at the individual level; therefore, a potential bias may exist between individual antibiotic use and the presence of resistance or the eradication of *H. pylori* in that individual. Parallel data on antibiotic resistance for each country during the study period were also unavailable. Nonetheless, as observed in other studies of infections similar to ours, [30, 31] resistance tends to follow antibiotic consumption patterns. Importantly, antibiotic sales records can provide an approximate measure of population‐level antibiotic pressure and how such consumption may influence the efficacy of empiric treatments that include the same antibiotic; however, this approach has inherent limitations, as it does not capture individual exposure histories, indication‐specific use, or fully reflect resistance patterns, and therefore should be interpreted cautiously as a surrogate for treatment efficacy. The small sample size in some countries and treatment regimens limited subgroup analysis, though results remained consistent when focusing on Spain and Italy. Additionally, generalizing our findings is challenging due to the limited number of cases and centers in many countries. The impact of prior antibiotic use on second‐line treatment was not assessed, and neither was antibiotic susceptibility (with genetic analyses or cultures). Furthermore, we did not differentiate types of macrolides, and the system could only model macrolide consumption, not that of other antibiotics like metronidazole or levofloxacin. However, resistance to metronidazole has been reported to have minimal impact on eradication success. We also acknowledge that key confounders such as smoking status, alcohol use, body mass index (BMI), and CYP2C19 metabolizer phenotype were not available in the registry and could not be included in the models. Lastly, as this was an ecological study, it linked community‐level macrolide use with individual outcomes, introducing a risk of ecological fallacy and limiting causal inference, as individual macrolide exposure was not measured. Additionally, potential confounders such as personal antibiotic history and regional resistance patterns may have influenced the overall findings.

The study strengths include the large sample size (27,549 patients), the long follow‐up (10‐year evaluation), the use of international antibiotic data, and the assessment of real‐world treatment effectiveness across multiple countries and common regimens.

In conclusion, prior clarithromycin exposure in the general population significantly reduces the efficacy of first‐line *H. pylori* clarithromycin‐containing eradication regimens, particularly when exposure occurred within the past 5 years and at higher doses. The most affected regimens were clarithromycin‐based triple therapies and certain bismuth‐based quadruple therapies containing clarithromycin. It is necessary for governments to develop and standardize mandatory reporting items to enable meaningful comparisons—such as treatment indications, treatment types, diagnostic methods, adverse effects, resistance rates, and others. Additionally, prospective longitudinal studies in large populations, including biological samples and extended follow‐up periods, are needed to establish the biological and epidemiological mechanisms underlying this association.

## Author Contributions

Olga P. Nyssen, Scientific Director and member of the project's Scientific Committee, planned and coordinated the study, designed and programmed the electronic case report form, extracted, analyzed, synthesized, and interpreted the data, wrote the first draft, acted as critical reviewer of the manuscript drafts, and approved the final submitted manuscript. Guillermo J. Ortega planned and coordinated the study, extracted, analyzed, synthesized, and interpreted the data, contributed to writing the first draft, and approved the final submitted manuscript. Leticia Moreira, Scientific Director and member of the project's Scientific Committee critically reviewed and approved the final submitted manuscript. Anna Cano‐Català, Pablo Parra, Francis Mégraud, and Colm O’Morain: members of the project's Scientific Committee, critically reviewed the manuscript drafts and approved the submitted manuscript. Luis Bujanda and Concepción Bravo‐Pache assisted with data interpretation, contributed to the writing of the first draft, and approved the submitted manuscript. Javier P. Gisbert, Principal investigator of the project, member of the Scientific Committee, obtained funding, designed the protocol and planned the study, analyzed and interpreted the data, collected patients, critically reviewed the manuscript drafts, and approved the final submitted manuscript. Laimas Jonaitis, Ángeles Pérez‐Aísa, Bojan Tepes, Pablo M. Wolfe Garcia, Perminder Singh Phull, Samuel J. Martínez Dominguez, Alfredo J. Lucendo, Javier Tejedor‐Tejada, Renate Bumane, Ana Garre, Jose M. Huguet, Monica Perona, Óscar Núñez, Manuel Pabón‐Carrasco, M. Castro‐Fernández, Miguel Areia, Jesús Barrio, Antonio Moreno Loro, Thomas J. Butler, María Soledad Marcos, Alma Keco‐Huerga, Manuel Domínguez Cajal, Maja Denkovski, Matteo Pavoni, György Miklós Buzás, Frode Lerang, Giuseppe Losurdo, Pablo M. Wolfe García, Samuel J. Martínez‐Domínguez, Juozas Kupcinskas, Mārcis Leja, Ricardo Marcos‐Pinto, Sinead M. Smith, Antonio Gasbarrini, Veronika Papp, Blas José Gómez Rodríguez, Mónica Sánchez Alonso, Ramón Pajares Villarroya, Pilar Pazo Mejide, Manuel Jiménez‐Moreno, Marta Pascual‐Mato, Milagrosa Montes, Leticia Moreira, Luis Bujanda, Javier P. Gisbert: acted as patients’ recruiters, critically reviewed the manuscript drafts, and approved the submitted manuscript.

## Funding

This work was supported by Horizon EUROPE Health, 101095359 UK Research and Innovation, 10058099 EU4Health, 101101252. The Hp‐EuReg project was promoted and funded by the European Helicobacter and Microbiota Study Group (EHMSG; www.helicobacter.org) and received support from the Spanish Association of Gastroenterology (AEG) and the Centro de Investigación Biomédica en Red de Enfermedades Hepáticas y Digestivas (CIBERehd). Hp‐EuReg was co‐funded by the European Union programme HORIZON (grant agreement number 101095359) and supported by the UK Research and Innovation (grant agreement number 10058099). Views and opinions expressed are however those of the author(s) only and do not necessarily reflect those of the European Union or the Health and Digital Executive Agency (HaDEA). Neither the European Union nor the granting authority can be held responsible for them. Hp‐EuReg was co‐funded by the European Union programme EU4Health (grant agreement number 101101252). Hp‐EuReg was also funded by Diasorin, Juvisé, and Biocodex; however, clinical data were not accessible to the companies, and they were not involved in any stage of the Hp‐EuReg study (design, data collection, statistical analysis, or manuscript writing).

## Ethics Statement

The Hp‐EuReg protocol was approved by the Ethics Committee of Hospital Universitario de la Princesa (Madrid, Spain), which acted as a reference Institutional Review Board (IRB, 20 December 2012), was conducted according to the guidelines of the Declaration of Helsinki, was classified by the Spanish Agency for Medicines and Medical Devices and was prospectively registered in Clinical Trials.gov under the code NCT02328131. Multinational ethics approvals were obtained at the country level (i.e., for each of the participating countries reported in Figure 2), with oversight coordinated through the Spanish reference IRB.

## Consent

This information is included in the text: “Written informed consent was obtained from all participants”.

## Conflicts of Interest

Javier P. Gisbert has served as speaker, consultant, and advisory member for or has received research funding from Mayoly, Allergan/Abbvie, Diasorin, Richen, Juvisé, and Biocodex. Olga P. Nyssen has served as a speaker or has received research funding from Mayoly and Allergan. The remaining authors declare no conflicts of interest.

## Supporting information


**Data S1:** Supporting Information.

## Data Availability

Data Transparency Statement: Raw data from the European Registry on *H. pylori* infection management (Hp‐EuReg) were generated at the Spanish Association of Gastroenterology–Research Electronic Data Capture. Derived data supporting the findings of this study are available from the first author and senior author (O.P.N. and J.P.G.) upon request. Data Sharing Statement: The data that support the findings of this study are not publicly available, since the information could compromise the privacy of research participants. However, data previously published on the Hp‐EuReg study, or de‐identified raw data referring to the current study, as well as further information on the methods used to explore the data, could be shared with no particular time constraint. Individual participant data will not be shared.
