# Supplementary material for: Long‐Term Effect of Macrolides on Helicobacter pylori Eradication: Data From the European Registry on Helicobacter pylori Management (Hp‐EuReg)
Source: Helicobacter. 2026 Feb 8;31(1):e70107. doi: 10.1111/hel.70107 (PMC12884029; doi:10.1111/hel.70107)
Supplement: Supplementary file 1 — Data S1: Supporting Information. [file HEL-31-e70107-s001.docx]

**SUPPLEMENTARY MATERIAL**

**File S1**. Hp-EuReg investigators

Jurij Bednarik, Department of Gastroenterology, Klinika Doktor 24 Ljubljana, SLOVENIA. Acquired data, critically reviewed the manuscript draft, and approved the submitted manuscript.

Eduardo Iyo, Department of Gastroenterology, Hospital Universitari Son Espases, Palma (Mallorca), SPAIN. Acquired data, critically reviewed the manuscript draft, and approved the submitted manuscript.

Fernando Bermejo, Department of Gastroenterology, Hospital Universitario de Fuenlabrada, Fuenlabrada, SPAIN. Acquired data, critically reviewed the manuscript draft, and approved the submitted manuscript.

Virginia Flores, Department of Gastroenterology, Hospital General Universitario Gregorio Marañón, Madrid, SPAIN. Acquired data, critically reviewed the manuscript draft, and approved the submitted manuscript.

Miguel Fernández-Bermejo, Department of Gastroenterology, Hospital Parque San Francisco, Cáceres, SPAIN. Acquired data, critically reviewed the manuscript draft, and approved the submitted manuscript.

Ian L. P. Beales, Norwich Medical School, University of East Anglia, Norwich, UNITED KINGDOM. Acquired data, critically reviewed the manuscript draft, and approved the submitted manuscript.

Alisan Kahraman, Gastroenterology and Hepatology Department, Max Grundig Clinic, Bühl/Baden, GERMANY. Acquired data, critically reviewed the manuscript draft, and approved the submitted manuscript.

Dan L. Dumitrascu, Iuliu Hatieganu University of Medicine and Pharmacy, Cluj-Napoca, ROMANIA. Acquired data, critically reviewed the manuscript draft, and approved the submitted manuscript.

Ana Beatriz Pozo Blanco, Department of Gastroenterology, Hospital Arnau Vilanova-Lliria, Valencia, SPAIN. Acquired data, critically reviewed the manuscript draft, and approved the submitted manuscript.

Enrique Alfaro, Department of Gastroenterology, Hospital de Barbastro, Barbastro, SPAIN. Acquired data, critically reviewed the manuscript draft, and approved the submitted manuscript.

Montserrat Planella, Department of Gastroenterology, Hospital Universitari Arnau de Vilanova, Institut de Recerca Biomèdica de Lleida (IRBLL), Lleida, SPAIN. Acquired data, critically reviewed the manuscript draft, and approved the submitted manuscript.

Victor A. Kamburov, Department of Gastroenterology, BalkanMed Medical Center, Sofia, BULGARIA. Acquired data, critically reviewed the manuscript draft, and approved the submitted manuscript.

Debora Compare, Department of Gastroenterology, Federico II University, Naples, ITALY. Acquired data, critically reviewed the manuscript draft, and approved the submitted manuscript.

Natasa Brglez Jurecic, Interni Oddelek, Diagnostic Centre, Bled, SLOVENIA. Acquired data, critically reviewed the manuscript draft, and approved the submitted manuscript.

Georges Kamtoh, Hepatic Medical, Private Medical Center, Krakow, POLAND. Acquired data, critically reviewed the manuscript draft, and approved the submitted manuscript.

Antonietta G. Gravina, Hepatogastroenterology Unit, University of study of Campania "L. Vanvitelli", Naples, ITALY. Acquired data, critically reviewed the manuscript draft, and approved the submitted manuscript.

Benito Velayos, Department of Gastroenterology, Hospital Clínico de Valladolid, Valladolid, SPAIN. Acquired data, critically reviewed the manuscript draft, and approved the submitted manuscript.

Eva Barreiro Alonso, Department of Gastroenterology, Hospital Central de Asturias (HUCA), Department of Pharmacology, Instituto de Investigación Sanitaria del Principado de Asturias (ISPA), Oviedo, SPAIN. Acquired data, critically reviewed the manuscript draft, and approved the submitted manuscript.

Sabina Hrubá, 1st internal clinic, Department of gastroenterology and hepatology, University hospital in Pilsen, Pilsen, CZECH REPUBLIC. Acquired data, critically reviewed the manuscript draft, and approved the submitted manuscript.

Teresa Angueira, Department of Gastroenterology, Hospital Universitario de Toledo, Toledo, SPAIN. Acquired data, critically reviewed the manuscript draft, and approved the submitted manuscript.

Piotr Szredzki, The John Paul II City Hospital, Rzeszow, POLAND. Acquired data, critically reviewed the manuscript draft, and approved the submitted manuscript.

Melanija Razov Radas, Department of Gastroenterology and Hepatology, General Hospital Zadar, Health Studies, University of Zadar, Zadar, CROATIA. Acquired data, critically reviewed the manuscript draft, and approved the submitted manuscript.

Consuelo Ramírez, Department of Gastroenterology, Hospital Universitari Arnau de Vilanova, Institut de Recerca Biomèdica de Lleida (IRBLL), Lleida, SPAIN. Acquired data, critically reviewed the manuscript draft, and approved the submitted manuscript.

Noelia Alcaide, Department of Gastroenterology, Hospital Clínico de Valladolid, Valladolid, SPAIN. Acquired data, critically reviewed the manuscript draft, and approved the submitted manuscript.

Christos Liatsos, Gastroenterology Department, 401 Military Hospital of Athens, Athens, GREECE. Acquired data, critically reviewed the manuscript draft, and approved the submitted manuscript.

Luis Fernández-Salazar, Department of Gastroenterology, Hospital Clínico de Valladolid, Medicine Department, School of Medicine, Universidad de Valladolid, Valladolid, SPAIN. Acquired data, critically reviewed the manuscript draft, and approved the submitted manuscript.

Pedro Delgado Guillena, Department of Gastroenterology, Hospital de Mérida, Mérida, SPAIN. Acquired data, critically reviewed the manuscript draft, and approved the submitted manuscript.

Sheyla Montori Pina, Department of Gastroenterology, Hospital Universitario de Navarra (HUN), Navarrabiomed, Universidad Pública de Navarra (UPNA), IdiSNA, Pamplona, SPAIN. Acquired data, critically reviewed the manuscript draft, and approved the submitted manuscript.

Piotr Eder, Department of Gastroenterology, Dietetics and Internal Medicine, Poznań University of Medical Sciences, Department of Gastroenterology, H. Święcicki University Hospital, Poznan, POLAND. Acquired data, critically reviewed the manuscript draft, and approved the submitted manuscript.

Jitka Vaculová, Department of Gastroenterology and Internal Medicine, University Hospital Brno, Faculty of Medicine, Masaryk University, Brno, CZECH REPUBLIC. Acquired data, critically reviewed the manuscript draft, and approved the submitted manuscript.

Jan Kral, Medic Kral s.r.o., Praha, CZECH REPUBLIC. Acquired data, critically reviewed the manuscript draft, and approved the submitted manuscript.

Enrique Montil Miguel, Department of Gastroenterology, Hospital Reina Sofia, Tudela, SPAIN. Acquired data, critically reviewed the manuscript draft, and approved the submitted manuscript.

Rosario Antón Ausejo, Department of Gastroenterology, Hospital Clínico Universitario de Valencia, Valencia, SPAIN. Acquired data, critically reviewed the manuscript draft, and approved the submitted manuscript.

Gema Gigante González de la Aleja, Department of Gastroenterology, Hospital Universitario de Toledo, Toledo, SPAIN. Acquired data, critically reviewed the manuscript draft, and approved the submitted manuscript.

Matteo Ghisa, Gastroenterology Unit, Department of Surgery, Oncology and Gastroenterology, University of Padua, Gastroenterology Unit, Department of Oncological Gastrointestinal Surgery, S. Maria del Prato Hospital, Padua, ITALY. Acquired data, critically reviewed the manuscript draft, and approved the submitted manuscript.

Henrique Fernandes-Mendes, Department of Gastroenterology, Centro Hospitalar Universitário de Santo António, ULS de Santo António, Porto, PORTUGAL. Acquired data, critically reviewed the manuscript draft, and approved the submitted manuscript.

Marinko Marušić, Department of Gastroenterology, University Hospital Sveti Duh, School of Medicine, University J. J. Strossmayer Osijek, Faculty of Health Studies, University of Rijeka, CROATIA. Acquired data, critically reviewed the manuscript draft, and approved the submitted manuscript.

Stergios N. Kouvaras, Refferal Endoscopy Unit, Private Endoscopy Unit, Halkida, GREECE. Acquired data, critically reviewed the manuscript draft, and approved the submitted manuscript.

Judith Gomez-Camarero, Department of Gastroenterology, Hospital Universitario de Burgos, Burgos, SPAIN Acquired data, critically reviewed the manuscript draft, and approved the submitted manuscript.

Wojciech Marlicz, Department of Gastroenterology, Pomeranian Medical University in Szczecin, The Centre for Digestive Diseases, Endoklinika, Szczecin, POLAND. Acquired data, critically reviewed the manuscript draft, and approved the submitted manuscript.

Deirdre McNamara, School of Medicine, Trinity College Dublin, Dublin, IRELAND. Acquired data, critically reviewed the manuscript draft, and approved the submitted manuscript.

Pilar Mata-Romero, Department of Gastroenterology, Hospital Universitario de Cáceres, Cáceres, SPAIN. Acquired data, critically reviewed the manuscript draft, and approved the submitted manuscript.

María Badía Martínez, Department of Gastroenterology, Hopital General de la Defensa, Department of Gastroenterology, Hospital Universitario Miguel Servet, Zaragoza, SPAIN. Acquired data, critically reviewed the manuscript draft, and approved the submitted manuscript.

Miguel Suárez Matías, Department of Gastroenterology, Hospital Virgen de la Luz, Cuenca, SPAIN. Acquired data, critically reviewed the manuscript draft, and approved the submitted manuscript.

Inmaculada Ortiz-Polo, Department of Gastroenterology, Hospital Universitario y Politécnico la Fe, Valencia, SPAIN. Acquired data, critically reviewed the manuscript draft, and approved the submitted manuscript.

Nayden Marinov Kandilarov, Aleksandrovska University Hospital, Sofia, BULGARIA. Acquired data, critically reviewed the manuscript draft, and approved the submitted manuscript.

Benito Hermida Pérez, Department of Gastroenterology, Hospital Valle del Nalón, Langreo, SPAIN. Acquired data, critically reviewed the manuscript draft, and approved the submitted manuscript.

Sotirios D. Georgopoulos, Athens Medical, P. Faliron General Hospital, Athens, GREECE. Acquired data, critically reviewed the manuscript draft, and approved the submitted manuscript.

Daniel Martin-Holgado, Department of Gastroenterology, Hospital Universitario de Cáceres, Cáceres, SPAIN. Acquired data, critically reviewed the manuscript draft, and approved the submitted manuscript.

Rosa Rosania, Department of Gastroenterology, Hepatology and Infectious Diseases, Otto von Guericke University Hospital, Magdeburg, GERMANY. Acquired data, critically reviewed the manuscript draft, and approved the submitted manuscript.

Alexander Link, Department of Gastroenterology, Hepatology and Infectious Diseases, Otto-von-Guericke University Magdeburg, Magdeburg, GERMANY. Acquired data, critically reviewed the manuscript draft, and approved the submitted manuscript.

Luis Hernández, Department of Gastroenterology, Hospital Santos Reyes, Aranda de Duero, SPAIN. Acquired data, critically reviewed the manuscript draft, and approved the submitted manuscript.

Mila Kovacheva-Slavova, Department of Gastroenterology, Queen Yoanna University Hospital, Medical University of Sofia, Sofia, BULGARIA .Acquired data, critically reviewed the manuscript draft, and approved the submitted manuscript.

Petra Čavajdová, University Hospital Hradec Kralove, Hradec Kralove, CZECH REPUBLIC. Acquired data, critically reviewed the manuscript draft, and approved the submitted manuscript.

Ramiro Carreño Macián, Department of Gastroenterology, Hospital Quirónsalud Vitoria, Vitoria, SPAIN. Acquired data, critically reviewed the manuscript draft, and approved the submitted manuscript.

Andreas Blesl, Department of Internal Medicine, Division of Gastroenterology and Hepatology, Medical University of Graz, Graz, AUSTRIA. Acquired data, critically reviewed the manuscript draft, and approved the submitted manuscript.

Guillem Soy, Department of Gastroenterology, Hospital Clínic de Barcelona, Institut Clínic de Malalties Digestives i Metabòliques (ICMDM), Barcelona, SPAIN. Acquired data, critically reviewed the manuscript draft, and approved the submitted manuscript.

Sergio Gil Rojas, Department of Gastroenterology, Hospital Virgen de la Luz, Cuenca, SPAIN. Acquired data, critically reviewed the manuscript draft, and approved the submitted manuscript.

David Přidal, Department of Gastroenterology, SPEA Olomouc, Olomouc, CZECH REPUBLIC. Acquired data, critically reviewed the manuscript draft, and approved the submitted manuscript.

Paola Chaudarcas, Department of Gastroenterology, Hospital Universitario Infanta Sofía, San Sebastián de los Reyes, SPAIN. Acquired data, critically reviewed the manuscript draft, and approved the submitted manuscript.

Giulia Fiorini, Cardiovascular Medicine Unit, Heart, Chest and Vascular Department, IRCCS Azienda Ospedaliero-Universitaria di Bologna, Hypertension and Cardiovascular Risk Research Center, Medical and Surgical Sciences Dept., Alma Mater Studiorum University of Bologna, Bologna, ITALY. Acquired data, critically reviewed the manuscript draft, and approved the submitted manuscript.

Pedro Almela, Department of Gastroenterology, Hospital General Universitario de Castellón, Ciencias de la Salud (Medicina), Universidad CEU Cardenal Herrera, Castellón, SPAIN. Acquired data, critically reviewed the manuscript draft, and approved the submitted manuscript.

Anna-Maria Tiefenthaller, Department of Gastroenterology, Barmherzige Schwestern Linz, Linz, AUSTRIA. Acquired data, critically reviewed the manuscript draft, and approved the submitted manuscript.

Lumir Kunovsky, 2nd Department of Internal Medicine - Gastroenterology and Geriatrics, University Hospital Olomouc, Faculty of Medicine and Dentistry, Palacky University Olomouc, Department of Surgery, University Hospital Brno, Faculty of Medicine, Masaryk University, Department of Gastroenterology and Digestive Endoscopy, Masaryk Memorial Cancer Institute, CZECH REPUBLIC. Acquired data, critically reviewed the manuscript draft, and approved the submitted manuscript.

Saioa De la Maza Ortiz, Department of Gastroenterology, Hospital Universitario de Basurto, Bilbao, SPAIN. Acquired data, critically reviewed the manuscript draft, and approved the submitted manuscript.

Cristina Suárez Ferrer, Department of Gastroenterology, School of Medicine, Universidad Autónoma de Madrid, Instituto de Investigación Hospital Universitario La Paz (IdiPAZ), Hospital Universitario La Paz, Madrid, SPAIN. Acquired data, critically reviewed the manuscript draft, and approved the submitted manuscript.

Antonio Cuadrado, Department of Gastroenterology and Hepatology, Hospital Universitario Marqués de Valdecilla, Clinical and Translational Research in Digestive Diseases, Valdecilla Research Institute (IDIVAL), Santander, SPAIN. Acquired data, critically reviewed the manuscript draft, and approved the submitted manuscript.

Francisco J. Rancel-Medina, Department of Gastroenterology, Complejo Asistencial Universitario de Palencia, Palencia, SPAIN. Acquired data, critically reviewed the manuscript draft, and approved the submitted manuscript.

Diego Burgos-Santamaría, Department of Gastroenterology and Hepatology, Hospital Universitario Ramón y Cajal, Madrid, SPAIN. Acquired data, critically reviewed the manuscript draft, and approved the submitted manuscript.

Suzanne Cauchi, Mater Dei Hospital, Msida, MALTA. Acquired data, critically reviewed the manuscript draft, and approved the submitted manuscript.

Isabel Pérez-Martínez, Department of Gastroenterology, Hospital Universitario Central de Asturias, Instituto de Investigación Sanitaria del Principado de Asturias (ISPA), Oviedo, SPAIN. Acquired data, critically reviewed the manuscript draft, and approved the submitted manuscript.

Daniel Abad, Department of Gastroenterology, Hospital Obispo Polanco, Department of Gastroenterology, Hospital Miguel Servet, Teruel, SPAIN. Acquired data, critically reviewed the manuscript draft, and approved the submitted manuscript.

Marko Nikolic, University Centre Sestre Milosrdnice, Zagreb, CROATIA. Acquired data, critically reviewed the manuscript draft, and approved the submitted manuscript.

Teresa Valdés-Lacasa, Department of Gastroenterology, Hospital Universitario Infanta Cristina, Parla, SPAIN. Acquired data, critically reviewed the manuscript draft, and approved the submitted manuscript.

Lara Gassner, Department of Gastroenterology, Landeskrankenhaus Salzburg, Universitätsklinik für Innere Medizin I, Salzburg, AUSTRIA. Acquired data, critically reviewed the manuscript draft, and approved the submitted manuscript.

Riccardo Vasapolli, Medical Department 2, University Hospital LMU Munich, Munich, GERMANY Acquired data, critically reviewed the manuscript draft, and approved the submitted manuscript.

Natalie Friedova, Department of Gastroenterology, Thomayer University Hospital, Prague, CZECH REPUBLIC. Acquired data, critically reviewed the manuscript draft, and approved the submitted manuscript.

Rosa M. Sáiz-Chumillas, Department of Gastroenterology, Hospital General de Almansa, Department of Gastroenterology, Complejo Hospitalario Universitario de Albacete, Almansa, SPAIN. Acquired data, critically reviewed the manuscript draft, and approved the submitted manuscript.

Tamara Matysiak-Budnik, Hepato-Gastroenterology & Digestive Oncology Unit, University Hospital of Nantes, Nantes, FRANCE. Acquired data, critically reviewed the manuscript draft, and approved the submitted manuscript.

Petr Bauer, Department of Gastroenterology, Hospital Děčín, Děčín, CZECH REPUBLIC. Acquired data, critically reviewed the manuscript draft, and approved the submitted manuscript.

Noa Kapteijn, Maag-Darm-Leverziekten, Erasmus MC, Rotterdam, THE NETHERLANDS. Acquired data, critically reviewed the manuscript draft, and approved the submitted manuscript.

Jan Křivinka, 2nd Department of Internal Medicine – Gastroenterology and Geriatrics, University Hospital Olomouc, Faculty of Medicine and Dentistry, Palacky University Olomouc, Olomouc, CZECH REPUBLIC. Acquired data, critically reviewed the manuscript draft, and approved the submitted manuscript.

Jorge Yebra Carmona, Department of Gastroenterology, Hospital Universitario de Móstoles, Móstoles, SPAIN. Acquired data, critically reviewed the manuscript draft, and approved the submitted manuscript.

Gustavo Óliver Patrón-Román, Department of Gastroenterology, Hospital Parque Llevant, Porto Cristo, SPAIN Acquired data, critically reviewed the manuscript draft, and approved the submitted manuscript.

Marino Venerito, Department of Gastroenterology, Hepatology and Infectious Diseases, University Hospital of Magdeburg, Magdeburg, GERMANY. Acquired data, critically reviewed the manuscript draft, and approved the submitted manuscript.

Manon C.W. Spaander, Department of Gastroenterology and Hepatology, Erasmus University Medical Center, Rotterdam, THE NETHERLANDS. Acquired data, critically reviewed the manuscript draft, and approved the submitted manuscript.

Carlos Rodríguez Pérez, Department of Gastroenterology, Hospital Universitario de Gran Canaria Doctor Negrín, Las Palmas de Gran Canaria, SPAIN. Acquired data, critically reviewed the manuscript draft, and approved the submitted manuscript.

Irene Blanco Bartolomé, Department of Gastroenterology, Hospital del Henares, Coslada, SPAIN. Acquired data, critically reviewed the manuscript draft, and approved the submitted manuscript.

Isabel Socorro Muñoz Hernández, Department of Gastroenterology, Hospital General Universitario Nuestra Señora del Prado, Talavera de la Reina, SPAIN. Acquired data, critically reviewed the manuscript draft, and approved the submitted manuscript.

Senador Moran Sanchez, Department of Gastroenterology, Servicio Murciano de Salud, Cartagena, SPAIN. Acquired data, critically reviewed the manuscript draft, and approved the submitted manuscript.

Jan Bornschein Nuffield Department of Experimental Medicine, University of Oxford, Oxford, UNITED KINGDOM. Acquired data, critically reviewed the manuscript draft, and approved the submitted manuscript.

Diego Ledro Cano, Department of Gastroenterology, Clínica HLA Santa Isabel, Department of Gastroenterology, Hospital Universitario Virgen Macarena, Sevilla, SPAIN. Acquired data, critically reviewed the manuscript draft, and approved the submitted manuscript.

Pilar Bernal Checa, Department of Gastroenterology, Hospital Universitario Miguel Servet, Instituto de Investigación Sanitaria de Aragón (IIS Aragón), Zaragoza, SPAIN. Acquired data, critically reviewed the manuscript draft, and approved the submitted manuscript.

Antonio M. Caballero-Mateos, Department of Gastroenterology, Hospital Universitario San Cecilio, Precision Medicine, Instituto de Investigación Biosanitaria de Granada, Granada, SPAIN. Acquired data, critically reviewed the manuscript draft, and approved the submitted manuscript.

Leticia Gimeno Pitarch, Department of Gastroenterology, Hospital General Universitario de Castellón, Castellón, SPAIN. Acquired data, critically reviewed the manuscript draft, and approved the submitted manuscript.

María de Lucas Gallego, Department of Gastroenterology, Hospital del Tajo, Aranjuez, SPAIN. Acquired data, critically reviewed the manuscript draft, and approved the submitted manuscript.

Jakub Langner, Beskydy Gastrocentre, Hospital Frydek-Mistek, Frydek-Mistek, CZECH REPUBLIC. Acquired data, critically reviewed the manuscript draft, and approved the submitted manuscript.

Ángela Martínez Herreros, Department of Gastroenterology, Hospital Universitario San Pedro, Logroño, SPAIN. Acquired data, critically reviewed the manuscript draft, and approved the submitted manuscript.

Patricia Sanz-Segura, Department of Gastroenterology, Hospital Royo Villanova, Zaragoza, SPAIN. Acquired data, critically reviewed the manuscript draft, and approved the submitted manuscript.

Melvyn Peña Gómez, Department of Gastroenterology, Hospital General de Valdepeñas, Ciudad Real, SPAIN. Acquired data, critically reviewed the manuscript draft, and approved the submitted manuscript.

Antonio Mestrovic, Department of Gastroenterology, University Hospital of Split, Split, CROATIA. Acquired data, critically reviewed the manuscript draft, and approved the submitted manuscript.

Gino Heeren, Department of Gastroenterology, Landeskrankenhaus Salzburg, Universitätsklinik für Innere Medizin I, Salzburg, AUSTRIA. Acquired data, critically reviewed the manuscript draft, and approved the submitted manuscript.

Adam Vasura, Department of Gastroenterology, Hepatology and Pancreatology, Internal and Cardiologic Clinic, University Hospital of Ostrava, Ostrava, CZECH REPUBLIC. Acquired data, critically reviewed the manuscript draft, and approved the submitted manuscript.

Patrick Dinkhauser, I. Interne Abteilung, Klinikum Wels-Grieskirchen, Wels, AUSTRIA. Acquired data, critically reviewed the manuscript draft, and approved the submitted manuscript.

Mirjana Kalauz, Endoscopy Unit, Division of Gastroenterology, Department of Internal Medicine, Clinical Hospital Center Zagreb, Internal Medicine, School of Medicine, University of Zagreb, Zagreb, CROATIA. Acquired data, critically reviewed the manuscript draft, and approved the submitted manuscript.

Maria de los Ángeles Mejías Manzano, Department of Gastroenterology, Hospital Virgen de Altagracia, Manzanares, SPAIN. Acquired data, critically reviewed the manuscript draft, and approved the submitted manuscript.

Petra Koňaříková, Tomas Bata Regional Hospital, Zlin, CZECH REPUBLIC. Acquired data, critically reviewed the manuscript draft, and approved the submitted manuscript.

Jose Xavier Segarra Ortega, Department of Gastroenterology, Hospital Universitario de Salamanca, Grupo de Investigación Salmantino en Aparato Digestivo (GISAD), Instituto de Investigación Biomédica de Salamanca, Salamanca, SPAIN. Acquired data, critically reviewed the manuscript draft, and approved the submitted manuscript.

Karin Steidl, Department of Internal Medicine, Barmherzige Brüder St. Veit/Glan, St. Veit an der Glan, AUSTRIA. Acquired data, critically reviewed the manuscript draft, and approved the submitted manuscript.

Alicia Granja Navacerrada, Department of Gastroenterology, Hospital Universitario de Fuenlabrada, Fuenlabrada, SPAIN. Acquired data, critically reviewed the manuscript draft, and approved the submitted manuscript.

Edel Berroa de la Rosa, Department of Gastroenterology, Hospital General Universitario Nuestra Señora del Prado, Department of Gastroenterology, Centro Médico De Diagnostico De Talavera, Talavera de la Reina, SPAIN. Acquired data, critically reviewed the manuscript draft, and approved the submitted manuscript.

Raquel García-Sánchez, Department of Gastroenterology, Hospital Universitario Infanta Leonor, Madrid, SPAIN. Acquired data, critically reviewed the manuscript draft, and approved the submitted manuscript.

Katja Repitsch, Department of Gastroenterology, Klinikum Klagenfurt am Wörthersee, Klagenfurt, AUSTRIA. Acquired data, critically reviewed the manuscript draft, and approved the submitted manuscript.

Antonio Díaz-Sánchez, Department of Gastroenterology, Hospital Universitario del Sureste, Arganda del Rey, SPAIN Acquired data, critically reviewed the manuscript draft, and approved the submitted manuscript.

Monika Šindlerová, Department of Gastroenterology, Fakultná Nemocnica Nitra, SLOVAKIA. Acquired data, critically reviewed the manuscript draft, and approved the submitted manuscript.

Jesus M. Gonzalez-Santiago, Department of Gastroenterology, Complejo Asistencial Universitario de Salamanca, Instituto de Investigación Biomédica de Salamanca (IBSAL), Centro de Investigación Biomédica en Red de Enfermedades Hepáticas y Digestivas (CIBERehd), Salamanca, SPAIN. Acquired data, critically reviewed the manuscript draft, and approved the submitted manuscript.

Martin Schnierer, Clinic of Internal Medicine, JFM CU Comenius University, Martin, SLOVAKIA. Acquired data, critically reviewed the manuscript draft, and approved the submitted manuscript.

Laura Larrey Ruiz, Department of Gastroenterology, Hospital Universitario Doctor Peset, Valencia, SPAIN. Acquired data, critically reviewed the manuscript draft, and approved the submitted manuscript.

Rodrigo Garcés-Durán, Department of Gastroenterology and Hepatology, Université catholique de Louvain, Cliniques Universitaires Saint-Luc, Brussels, BELGIUM. Acquired data, critically reviewed the manuscript draft, and approved the submitted manuscript.

Jesús Daniel Fernández-de Castro, Department of Gastroenterology, Complexo Hospitalario Universitario de Ourense, Ourense, SPAIN. Acquired data, critically reviewed the manuscript draft, and approved the submitted manuscript.

Irene Arteagoitia, Department of Gastroenterology, Hospital de Cruces, Barakaldo, SPAIN. Acquired data, critically reviewed the manuscript draft, and approved the submitted manuscript.

Maria Fraile Gonzalez, Department of Gastroenterology, Hospital Universitario San Pedro, Logroño, SPAIN. Acquired data, critically reviewed the manuscript draft, and approved the submitted manuscript.

Theodore Rokkas, Department of Gastroenterology, Henry Dunant Hospital, Athens, GREECE. Acquired data, critically reviewed the manuscript draft, and approved the submitted manuscript.

Pierre Ellul, Department of Gastroenterology, Mater Dei Hospital, Msida, MALTA. Acquired data, critically reviewed the manuscript draft, and approved the submitted manuscript.

Lyudmila Boyanova, Department of Medical Microbiology, Medical University of Sofia, Sofia, BULGARIA. Acquired data, critically reviewed the manuscript draft, and approved the submitted manuscript.

Antonia Perelló, Department of Gastroenterology, Hospital Universitari Son Espases, Palma (Mallorca), SPAIN. Acquired data, critically reviewed the manuscript draft, and approved the submitted manuscript.

Eduardo Albéniz, Department of Gastroenterology, Hospital Universitario de Navarra (HUN), Navarrabiomed, Universidad Pública de Navarra (UPNA), IdiSNA, Pamplona, SPAIN. Acquired data, critically reviewed the manuscript draft, and approved the submitted manuscript.

**Figure S1**. Sensitivity analyses of the effect of macrolide community consumption on clarithromycin-based treatment effectiveness for different delay periods between consumption and treatment. Different consumption levels (0.6, 2, 4, 6, 8 DDD/1000 inhabitants/day) are color-coded. The x-axis represents delay times between consumption and treatment year.

**(A) excluding Spain**


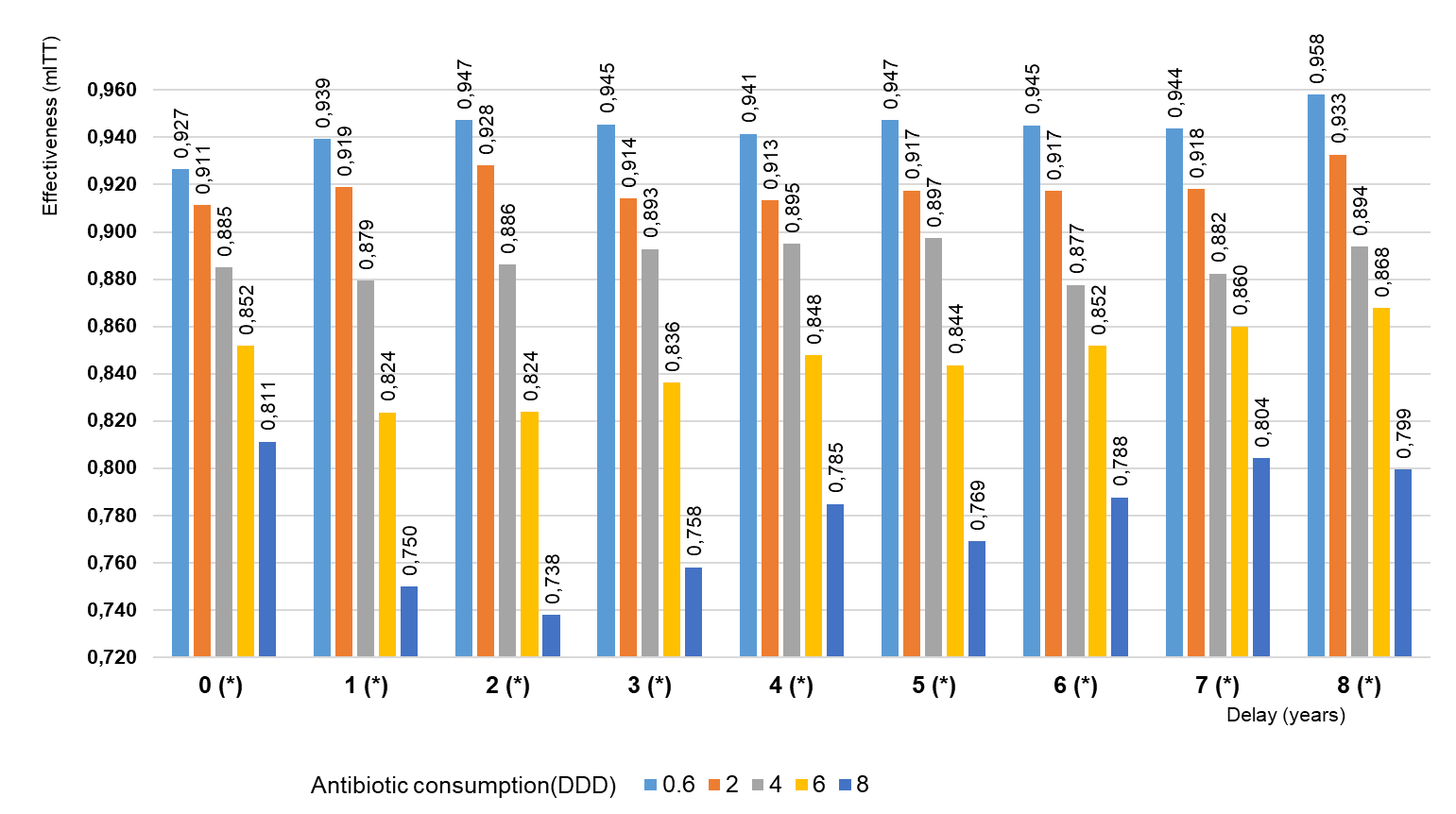


Treatment effectiveness was determined by modified intention to treat (mITT) analysis. *Significant model improvement with a likelihood ratio test (LRT), p<0.05. DDD: defined daily dose.

**(B) excluding Spain & Italy**


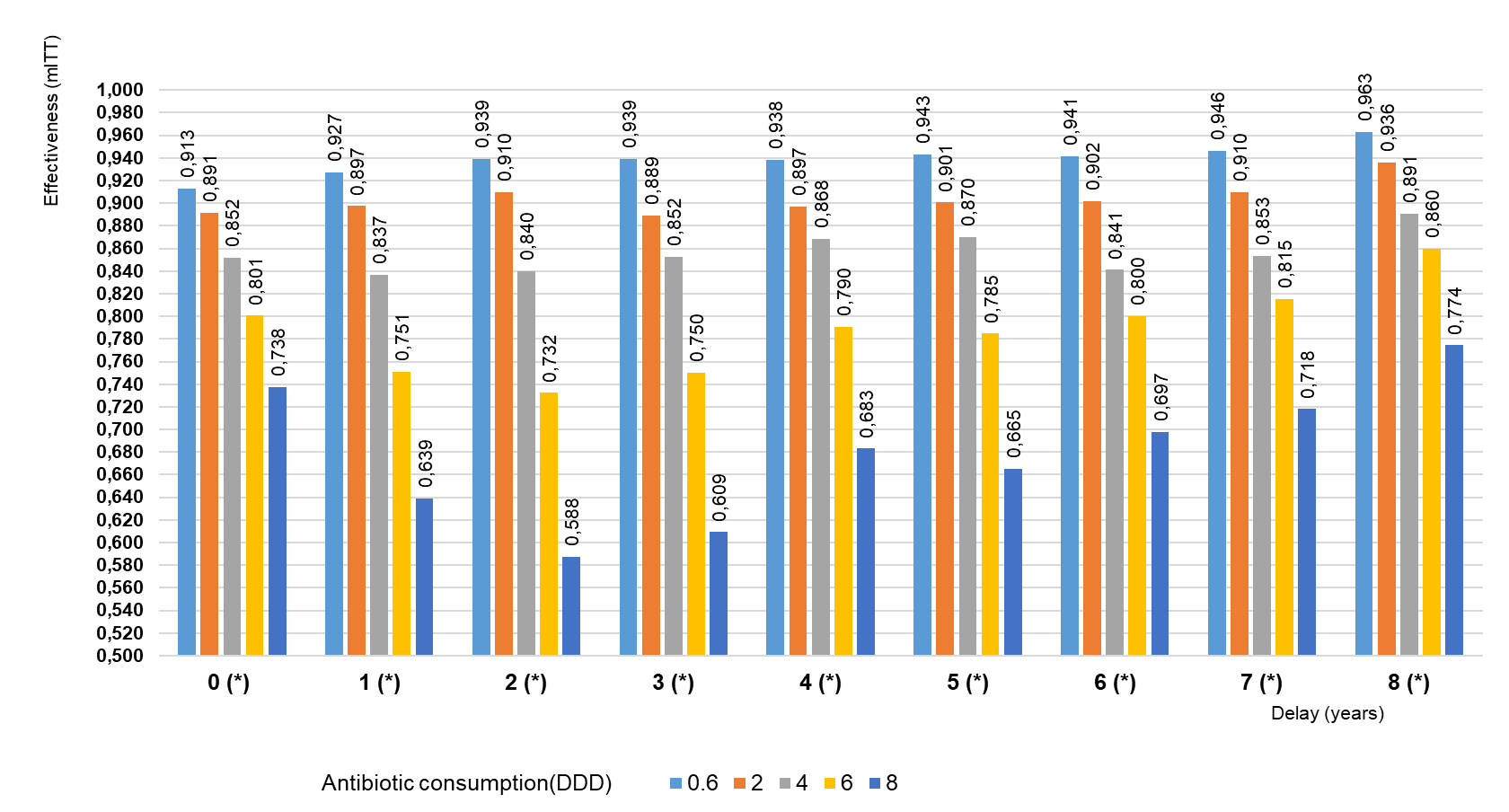


Treatment effectiveness was determined by modified intention to treat (mITT) analysis. *Significant model improvement with a likelihood ratio test (LRT), p<0.05. DDD: defined daily dose.

**(C) excluding Spain & Italy & Slovenia**


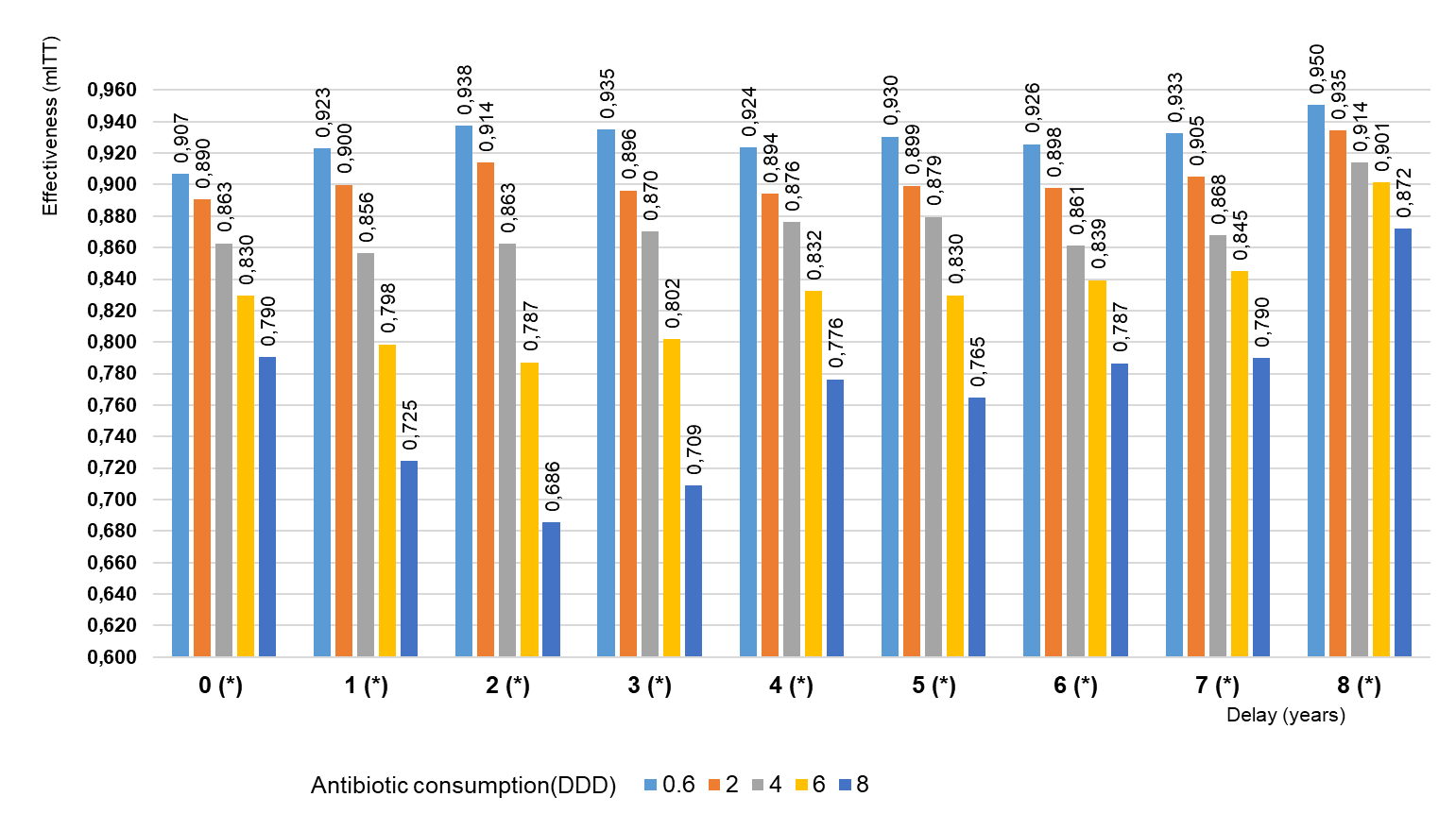


Treatment effectiveness was determined by modified intention to treat (mITT) analysis. *Significant model improvement with a likelihood ratio test (LRT), p<0.05. DDD: defined daily dose.
